# Preparation of High Strength Plywood from Partially Delignified Densified Wood

**DOI:** 10.3390/polym12081796

**Published:** 2020-08-11

**Authors:** Matthias Jakob, Gregor Stemmer, Ivana Czabany, Ulrich Müller, Wolfgang Gindl-Altmutter

**Affiliations:** Department of Material Sciences and Process Engineering, Institute of Wood Technology and Renewable Materials, University of Natural Resources and Life Sciences (BOKU), Konrad Lorenz-Strasse 24, 3430 Tulln, Austria; hnr@boku.ac.at (G.S.); ivana.czabany@boku.ac.at (I.C.); ulrich.mueller@boku.ac.at (U.M.); wolfgang.gindl-altmutter@boku.ac.at (W.G.-A.)

**Keywords:** chemical treatment, delignification, densification, plywood, wood composite, wood compression, wood modification, wood products

## Abstract

Wood and natural fibers exhibit an advantageous combination of good mechanics at comparably low density. Nevertheless, comparing absolute strength and stiffness, wood is clearly inferior to materials such as metals and engineered composites. Since there is a strong correlation between wood density and wood mechanical performance, densification by transversal compression suggests itself as a route towards improved mechanics. Partially delignified densified spruce veneers with excellent tensile properties were produced by means of an alkaline (AL) and an organosolv (OS) approach. Plywood specimens were manufactured using treated veneers glued with a phenol-resorcinol-formaldehyde adhesive and were compared with plywood samples made of native spruce veneers (Ref) and spruce veneer densified after plasticization by water impregnation (H_2_O). Roughly, the bending strength and the modulus of elasticity of plywood from partially delignified densified wood were improved by a factor of 2.4 and 3.5, respectively. Interlaminar shear strength did not match this improvement after partial delignification. Together with excessive thickness swelling, this might be a drawback of partially delignified densified wood in need for further research.

## 1. Introduction

Wood and natural fibers exhibit an advantageous combination of good mechanics at comparably low density, which makes them ideal construction materials [1,2]. These favorable features have been valued by engineers for hundreds of years. One of the most outstanding technological achievements in the human history, namely the ability to overcome gravity and successfully construct an airplane, would not have been possible without using wood and natural fibers. At the beginning of aeronautical engineering, wood and natural fibers were the only materials being strong enough to withstand flight loads and light enough to make flying possible. In the late 1930s, a new era in the development of construction materials began, and the first all-metal airplanes were manufactured [3]. Composites made of man-made fibers such as glass- or carbon-fibers and petroleum-based matrix materials followed. Although these materials are highly favorable due to their excellent mechanical properties at low density, their production has a tremendous influence on the environment. Beside the high energy consumption during their production, these materials are mostly fossil-based and the resulting waste is difficult to handle [4].

For environmental protection, concepts such as the end of life vehicles directive of the EU from the year 2000 has been developed [5]. The fact that natural fibers are renewable, biodegradable and possess a small carbon footprint, a low environmental impact and good mechanical properties, make them attractive construction materials for cars and airplanes to meet the requirements of the EU directive. Moreover, their comparably low density enables the reduction in weight and thus improves fuel efficiency [4,5,6]. This in turn can help to follow the Kyoto protocol [5]. Thus, these days more and more engineers are working on composites made of natural fibers [7,8,9]. Although natural fibers possess good specific strength and stiffness, there are still several drawbacks of these materials. Beside poor compatibility with several polymeric matrices and high moisture sensitivity [10], it is very difficult to produce a structural component with high reliability due to a high inherent variability of the mechanical properties of these annual plant fibers [11]. The high variability is even more pronounced due to the use of mechanical fractionation that leads to the formation of micro cracks in the fiber structure [12]. Finally, long term availability and additional problems such as fogging or odor of fibers also have to be considered [5].

Unlike annual natural fibers, wood is available throughout the year. Moreover, wood offers the possibility to manufacture a wide range of different wood-based materials [13]. An advanced degree of homogenization of the mechanical performance of wood can be achieved with increasing comminution. However, a significant drawback of the homogenization process is the loss of the highly favorable structural directionality and the resulting reduction in strength [14]. Therefore, the most promising material for structural applications would be a composite made of highly homogenized wood in which its prominent structural directionality would be preserved. Disregarding environmental advantages, wood is clearly inferior to well-established structural materials such as metals or polymer composites in terms of absolute strength and stiffness [2]. In order to increase the competitiveness of wood in this field of application, an improvement of absolute mechanical performance is thus highly desirable. Densification by transversal compression is a well-known strategy to enhance the mechanical properties of wood-based materials and has been known for decades [15,16,17,18].

Recently two papers were published in which wood densification following the extraction of non-cellulosic wood polymers, in particular lignin, was addressed. Using this treatment step, very significant improvements in mechanical performance were achieved after densification [19,20]. Yano and co-workers already recognized the potential of delignified, compressed wood around 20 years ago [21,22]. Since then, different processes have been developed to extract non-cellulosic polymers (e.g., treatment with acid [20,23], base [19] or a combination of them [21,24]). Song, et al. [19] suggest that the extraction of amorphous polymers results in an enrichment of free hydroxyl groups at the cellulose microfibrils which can build additional inter- and intramolecular hydrogen-bonds when pressed together and hence increase the mechanical properties.

While past studies focused mainly on single layers of densified wood, the present work evaluates the feasibility of high-performance plywood based on partially delignified spruce. In particular, the absolute performance of plywood as well as differences in properties of densified wood pre-treated with two different delignification methods (alkali; organosolv) are examined.

## 2. Materials and Methods

### 2.1. Delignification Protocols

Defect-free sliced spruce wood veneer with a nominal thickness of 1.4 mm (tangential) and a density of 0.39 g cm^−3^ was used as raw material and also served as reference samples (Ref). In order to test the potential of the treatments on an industrial scale, no specific requirements were set for the veneers. The sample size was 50 mm × 50 mm. Two delignification protocols were carried out: (1) In an organosolv (OS) protocol, the specimens were first soaked in a 2:1 (*v*/*v*) mixture of ethanol (EtOH) (Carl Roth GmbH + Co. KG, Karlsruhe, Germany) and deionized water (DI) until they drowned. To accelerate the process, vacuum was applied by using a desiccator and a vacuum pump. Subsequently, specimens were immersed in a 2:1 (*v*/*v*) mixture of EtOH and DI, containing 1.5% (*v*/*v*) acetic acid (HAc) (Carl Roth GmbH + Co. KG, Karlsruhe, Germany) and placed in a 5 L pressure reactor (Büchiglas, Uster, Switzerland). Delignification was carried out at 170 °C and 14 bar for 180 min. The heating-up time was 75 min and the cooling time was 120 min. No stirring was performed in order to avoid damage to the veneer specimens. (2) In an alkaline (AL) protocol, the specimens were primarily impregnated in DI until they drowned. This process was again accelerated by applying vacuum. Afterwards, the veneers were put in an aqueous solution, based on the publication of Song, et al. [19], of mixed 0.4 mol L^−1^ sodium sulphite (Na_2_SO_3_) (Carl Roth GmbH + Co. KG, Karlsruhe, Germany) and 2.5 mol L^−1^ sodium hydroxide (NaOH) (Carl Roth GmbH + Co. KG, Karlsruhe, Germany). By means of a water bath the temperature of the solution was kept at 98.5 °C for 240 min at atmospheric pressure. Glass lids were placed onto the containers in order to prevent excessive evaporation of the reaction solution. For both methods, the conditions chosen were derived from screening experiments with varying severity. As a target, specimens delignified to a high softness and flexibility, but with well-maintained structural integrity in terms of easy manipulation without splitting or fibrillation were deemed appropriate. After delignification, the specimens were at pH 3.5 (OS) and pH 14 (AL), respectively. Washing was carried out in DI at 60 °C until the washing water reached a stable pH of 7. For reference purposes in addition to Ref veneer, veneer slices were also soaked in deionized water at 98.5 °C for 4 h in order to achieve complete saturation (H_2_O).

### 2.2. Densification Protocol

After delignification (AL, OS) or saturation with water (H_2_O), the specimens were densified in a hydraulic hot press (UK 300 KN, F. u. A. Arnold Maschinenbau, Vienna, Austria). At 120 °C a maximum pressure of 20 MPa was built-up during 15 min in order to progressively remove water from the fully swollen specimens. Thereafter, the pressure was maintained overnight at room temperature. Finally, the densified specimens were stored in a climate chamber kept at 20 °C and 65% relative humidity until further use.

### 2.3. Preparation of Densified Plywood

The equilibrated specimens were coated with 400 g m^−2^ phenol-resorcinol-formaldehyde adhesive (Aerodux 185, Dynea AS, Krems, Austria) and assembled into cross-laminated stacks of plywood with the grain orientation sequence 0°-90°-0° for the uncompressed reference and 0°-90°-0°-90°-0° for the densified variants. Adhesive bonding was again carried out in a hydraulic hot press at 80 °C and a pressure of 1.5 MPa. All specimens were pressed for at least 12 h, ensuring a curing time well above the requirements given by the adhesive manufacturer. Thereafter, the plywood specimens were left again to equilibrate in a climate chamber at 20 °C and 65% rel. humidity. An additional set of plywood specimens with 7 layers for the untreated reference, and 11 layers for the densified variants was produced using an identical approach. These specimens with high thickness and comparably short length were produced to provoke interlaminar shear failure during 3-point bending tests.

### 2.4. Characterization

The mass loss of the densified chemically treated specimens was calculated as:

(1) $$ML\;\left(\%\right)=\frac{\left(m_{un}-m_{tr}\right)}{m_{un}}\times 100$$

where *ML* is the mass loss of the chemically treated specimens, *m_un_* is the mass of oven-dried untreated specimens and *m*_tr_ is the mass of oven-dried chemically treated specimens.

Acid insoluble lignin content was determined according to TAPPI standard T 222 om-02 [25] on untreated and uncompressed chemically treated specimens. An approximate assignment of the total mass loss into mass loss due to Klason lignin content reduction and hemicelluloses elution was carried out using a simple balance based on the following equations under the simplifying assumption that no cellulose degradation occurred:

(2) $$ML_{kl}\;\left(\%\right)=KL_{un}-\left(\left(100-ML\right)\times \frac{KL_{tr}}{100}\right)$$

where *ML_kl_* is the mass loss due to Klason lignin delignification, *KL_un_* is the Klason lignin content of the untreated specimens, *ML* is the mass loss of the chemically treated specimens, *KL_tr_* is the Klason lignin content of the chemically treated specimens

(3) $$ML_{h}\;\left(\%\right)=ML-ML_{kl},$$

and *ML_h_* is the mass loss due to hemicelluloses degradation.

The estimated new chemical composition of the chemically treated specimens was calculated under the assumption, that nearly no cellulose degradation occurred:

(4) $$H_{tr}\;\left(\%\right)=\frac{\left(H_{un}-ML_{h}\right)}{\left(100-ML\right)}\times 100$$

where *H_tr_* is the hemicelluloses content of the chemically treated specimens, *H_un_* is the hemicelluloses content based on Fengel and Wegener [26], *ML_h_* is the mass loss due to hemicelluloses degradation, *ML* is the mass loss of the chemically treated specimens

(5) $$C_{tr}\;\left(\%\right)=100-KL_{tr}-H_{tr},$$

and *C_tr_* is the cellulose content of the chemically treated specimens, *KL_tr_* is the Klason lignin content of the chemically treated specimens and *H_tr_* is the hemicelluloses content of the chemically treated specimens.

Light microscopy (Axioplan 2 Imaging, Carl Zeiss GmbH, Vienna, Austria) was performed with wood specimens embedded into epoxy resin (Agar Low Viscosity Resin Kit, Agar Scientific Ltd., Stansted, UK) and sectioned with a diamond knife (trim 45, Diatome Ltd., Nidau, Switzerland).

The tensile properties of the veneers were determined along their grain direction using a universal testing machine (Zwick-Roell 20 kN, ZwickRoell, Ulm, Germany) equipped with a 20 kN load cell (ZwickRoell, Ulm, Germany). The strain was measured via the crossbeam travel. Specimens with a length of 50 mm and a width of 10 mm were cut by means of a circular saw and fixed to the grips of the tensile testing machine. The specimens were strained at a rate of 1 mm min^−1^ until failure. The tensile strength and the tensile modulus of elasticity were calculated based on F_max_ and on the slope between 10 and 40% of F_max_, respectively.

Three-point bending tests along the grain direction of the top veneer layer were conducted with plywood specimens using the same testing machine. The strain was measured via the crossbeam travel. For specimens with a length of 50 mm the tested free length was 45 mm and the deformation rate was 5 mm min^−1^. The bending strength and the bending modulus of elasticity were calculated based on F_max_ and on the slope between 10 and 40% of F_max_, respectively.

Dimensional stability of the plywood specimens was determined in the out-of-plane direction by means of complete immersion in 20 °C DI for 24 h. The thickness swelling was calculated as:

(6) $$\Delta T\;\left(\%\right)=\frac{\left(T_{t}-T_{0}\right)}{T_{0}}\times 100$$

where Δ*T* is the thickness swelling, *T_t_* is the thickness of the specimens after immersion and *T*_0_ is the initial thickness of the specimens.

## 3. Results and Discussion

All results shown represent arithmetic mean with standard deviation. While a simple immersion in deionized water (H_2_O) did not result in a measurable change of dry mass, the delignification procedures chosen for this study resulted in different dry mass loss, but fairly similar Klason lignin content as shown in Table 1. While the acid-insoluble lignin content was reduced to approximately 2/3 of the native content, the total dry mass loss exceeded the mass loss due to delignification. It is assumed that besides lignin, a substantial amount of hemicelluloses was also extracted during treatments. The chemical composition of European spruce wood is approximately 40.4% cellulose, 31.1% hemicelluloses, 28.2% lignin and 0.3% extractives and ash [26]. According to these values and the simplifying assumption of no cellulose degradation, an allocation of fractions of mass loss to lignin and hemicelluloses as shown in Table 1 was carried out. We assume that the mass loss due to the delignification treatments AL (18.4%) and OS (26.3%) is the sum of roughly 11.4% and 15% mass loss due to Klason lignin delignification and 7% and 11.3% mass loss due to hemicelluloses extraction, respectively. As a result of delignification, the relative cellulose content increases in treated specimens as shown in Table 2, showing the chemical composition of Ref samples according to Fengel and Wegener [26] and the estimated composition of chemically treated veneers of this study. As already mentioned, the acid-insoluble lignin content was reduced to approximately 2/3 of the native content. The hemicelluloses content was reduced by roughly 5% and 14% by AL and OS treatment, respectively. The reduction of these polymers resulted in an increase of the cellulose content to approximately 49.5% for AL- and 54.8% for OS-treated specimens.

**Figure 1:**
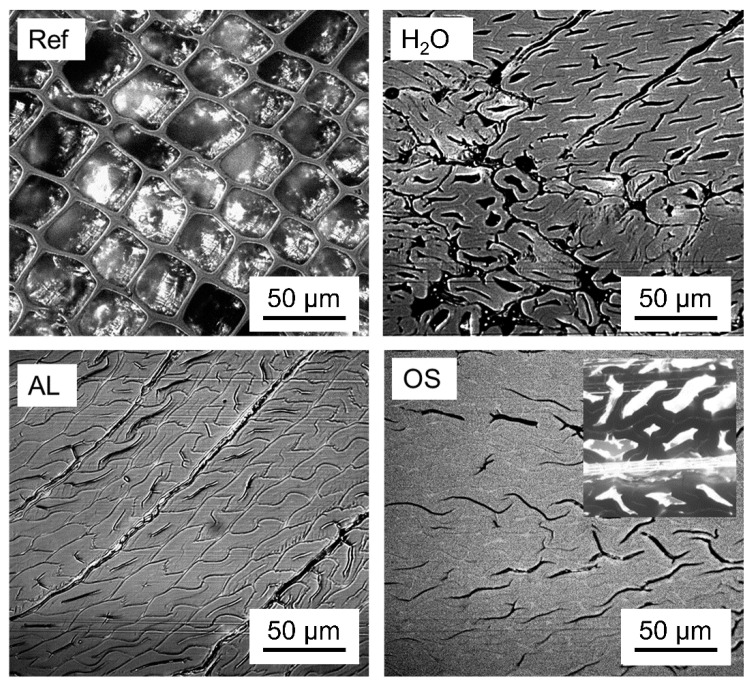
Representative incident light microscopy of untreated reference samples (Ref), samples densified after soaking with water (H_2_O), densified after alkali-delignification (AL), and densified after organosolv delignification (OS); Inset shows incomplete collapse of earlywood.

Densification of the chemically treated veneers led to similar results in thickness for both procedures, with density increasing by a factor of 2.46 for the AL and 2.1 for the OS variant as shown in Table 3. By comparison, the densification of specimens plasticized by treatment with water (H_2_O) was slightly less efficient with a factor 2.0. As shown in Figure 1, densification results in extensive cell wall collapse throughout the latewood region of the annual rings. The inset in the image displaying the OS variant shows incomplete collapse found in earlywood. This phenomenon can be explained by the anatomical structure of wood. In this work, wood veneers were compressed in tangential direction. Latewood and earlywood cells start to collapse at the same time, if densification occurs in tangential direction. Due to the thicker walls, cell closure appears first in latewood. After closure, latewood regions act as reinforcement columns embedded in earlywood regions until they start to buckle into the earlywood [27,28]. Latewood buckling ends with a readjustment of the latewood/earlywood interaction and leads to a new and more stable structure which can resist additional loads without extended compression [28]. Also the H_2_O variant, which was not delignified, showed extensive cell collapse, but also separation of individual cells and cell aggregates (Figure 1).

**Figure 2:**
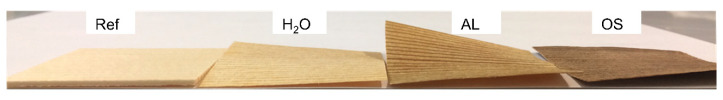
Representative photographs of single veneers in untreated condition (Ref), densified after soaking with water (H_2_O), densified after alkali-delignification (AL), and densified after organosolv delignification (OS).

As shown in Figure 2, densified veneers warped compared to untreated Ref veneers after conditioning at 20 °C and 65% relative humidity. It is assumed, that the earlywood/latewood readjustment is the reason for veneers warping. The delignified variants AL and OS also showed pronounced darkening associated with chemical treatment and ensuing change in wood chemistry.

**Figure 3:**
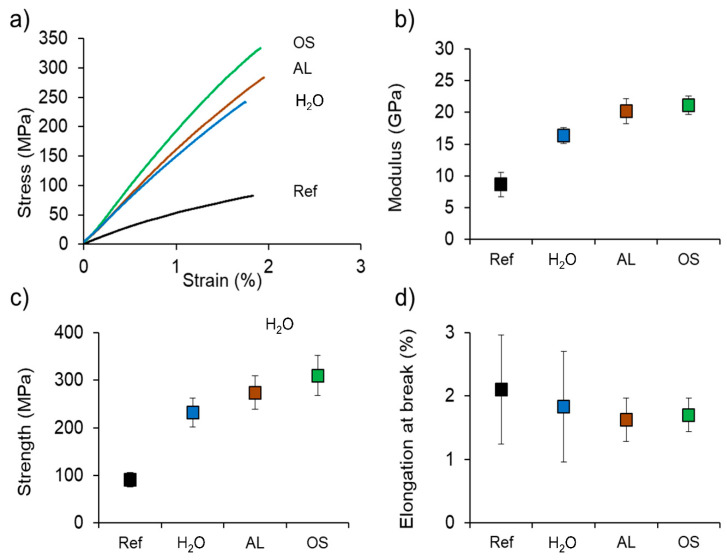
Mechanical characterization of reference (Ref), densified water-soaked (H_2_O), densified alkali-treated (AL) and densified organosolv-treated (OS) spruce veneers conditioned at 20 °C and 65% relative humidity by longitudinal tensile tests: (**a**) Representative stress-strain curves for each treatment; (**b**) Elastic Modulus; (**c**) Strength; (**d**) Elongation at break. (*n* = 15 for Ref; *n* = 10 for H_2_O; *n* = 10 for AL; *n* = 10 for OS).

Figure 3 shows the results of the mechanical characterization of non-treated and treated veneers by tensile tests. Stress-strain curves (Figure 3a) reflect the enormous impact of treatments on the tensile properties. According to Figure 3b, the mean modulus of elasticity increased by the factor of 2.34 and 2.44 for compressed AL- (20.23 ± 1.97 GPa) and OS-treated (21.16 ± 1.44 GPa) specimens, respectively, compared to the Ref (8.66 ± 1.90 GPa) specimens. Notably, these increases exceed the improvement by the factor 1.89, which was achieved by densification without prior delignification (variant H_2_O, 16.38 ± 1.23 GPa). The comparative data of tensile strength of Ref and densified AL- and OS-treated veneers parallel-to-grain direction are shown in Figure 3c. The mean tensile strength increased by the factor of 3.01 and 3.40 for densified AL- (273.95 ± 35.51 MPa) and OS-treated (309.60 ± 42.24 MPa) specimens, respectively, compared to the Ref (90.97 ± 14.77 MPa) specimens. Again, delignification prior to densification proves more effective than densification alone, where an improvement by a factor of 2.55 was observed (variant H_2_O, 232.38 ± 30.05 MPa).

A similar increase in tensile strength of chemically pre-treated specimens (~270 MPa) was obtained by Frey, et al. [20] who used an acidic delignification protocol on Norway spruce samples. This treatment resulted in a complete delignification of the specimens and delivered a high modulus of elasticity up to values of 35 GPa. Very efficient delignification and densification of different wood species resulted in record improvements of mechanical performance by up to a factor of 5 [19]. These improvements exceed pure scaling with increasing density. A comparison of the increase factors of the density (AL: 2.46 and OS: 2.1) with the increase factors of the tensile strength (AL: 3.01 and OS: 3.40) shows that the improvement in strength observed in the present study was not only density-dependent. It is proposed that additional hydrogen bond formation, enabled due to lignin extraction, is the reason for higher strength increase [19]. Furthermore, densification probably leads to mechanical interlocking at the micro- and nano-scales, which may benefit mechanical performance. Finally, delignification and removal of hemicelluloses result in a net increase of the fraction of cellulose in densified wood. As cellulose is per se significantly stronger and stiffer than the other cell wall polymers [29,30], an increase in mechanical performance may be expected. Although the absolute tensile properties of the specimens used in this study have been increased, they are still at a disadvantage compared to flax, hemp and jute which are typical fibers used in natural fiber composites (NFCs). Nevertheless, if the factor density is excluded and the specific tensile strength (AL: 285 MPa cm^3^ g^−1^ and OS: 370 MPa cm^3^ g^−1^) is calculated, the presented specimens can compete with e.g., hemp (214–581 MPa cm^3^ g^−1^) [31].

**Figure 4:**
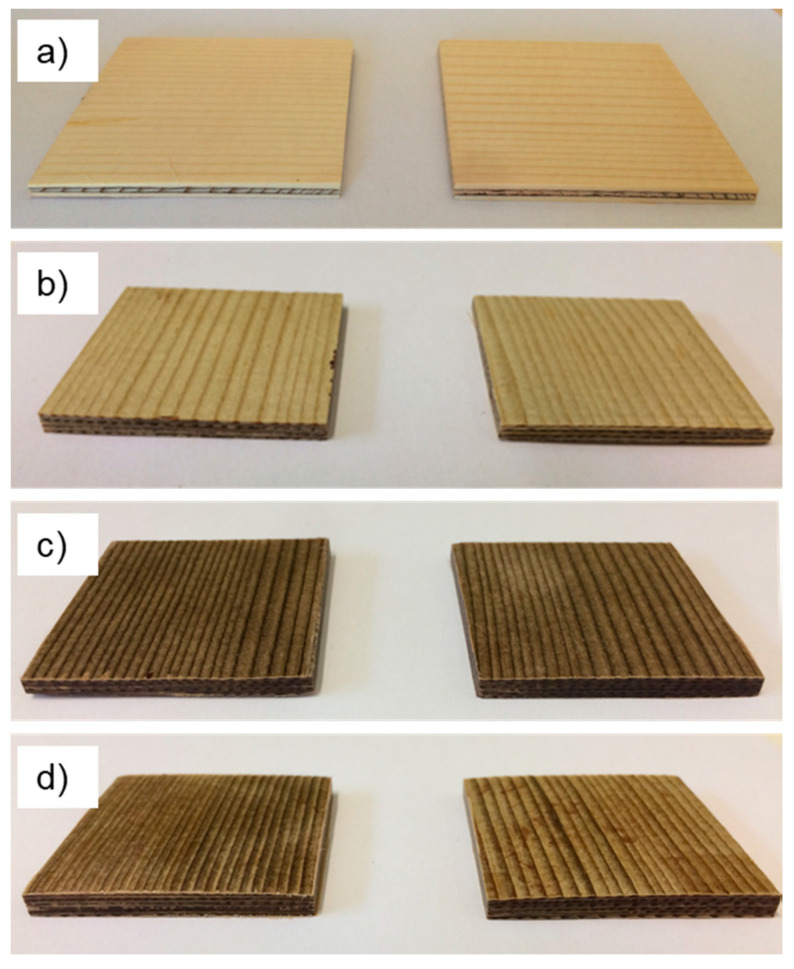
Representative photographs of plywood samples: (**a**) Made of reference veneers; (**b**) Made of densified water-soaked veneers; (**c**) Made of densified organosolv-treated veneers; (**d**) Made of densified alkali-treated veneers.

Figure 4a shows plywood made of Ref plies and Figure 4b–d show plywood made of densified water-soaked, as well as OS- and AL-treated plies, respectively.

**Figure 5:**
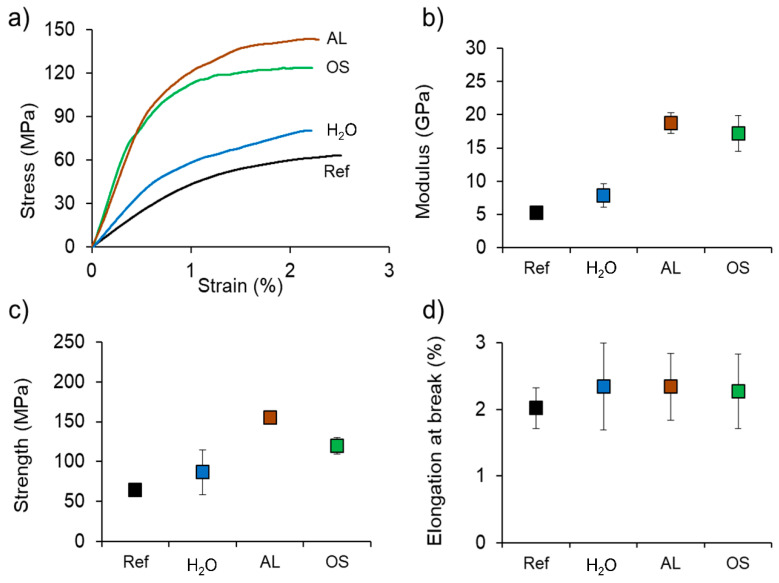
Mechanical characterization of plywood samples made of reference (Ref), densified water-soaked (H_2_O), densified alkali-treated (AL) and densified organosolv-treated (OS) spruce veneers conditioned at 20 °C and 65% relative humidity by longitudinal bending tests: (**a**) Representative stress-strain curves for each treatment; (**b**) Elastic Modulus; (**c**) Strength; (**d**) Elongation at break. (*n* = 8 for Ref; *n* = 8 for H_2_O; *n* = 8 for AL; *n* = 8 for OS).

Figure 5 shows the findings of the corresponding mechanical characterization of the plywood samples by bending test. The stress-strain diagram based on the mean values shown in Figure 5a reveals different performance of the four plywood variants produced. Especially the steep curves of the chemically treated specimens AL and OS are of high interest compared to the Ref and the H_2_O specimens. They indicate that the elastic modulus increased, which can also be seen in Figure 5b. While H_2_O (7.85 ± 1.78 GPa) specimens showed an increase by a factor 1.50, the mean modulus of elasticity shown in Figure 5b increased by the factor of 3.57 and 3.28 for compressed AL- (18.71 ± 1.56 GPa) and OS-treated (17.21 ± 2.71 GPa) specimens, respectively, compared to the Ref (5.24 ± 0.52 GPa) specimens. The mean bending strength seen in Figure 5c increased by the factor of 2.42 and 1.87 for densified AL- (155.05 ± 6.80 MPa) and OS-treated (119.76 ± 10.23 MPa) specimens, respectively, compared to the Ref (64.18 ± 5.67 MPa) specimens. Again, the increase observed for H_2_O (86.81 ± 28.05 MPa) specimens was more modest with a factor of 1.35. There is no report on plywood made of partially delignified densified veneers, to our knowledge. Others have shown that the compression of veneers alone, without prior delignification, can already improve the bending properties of plywood [32,33]. Bekhta, et al. [32] manufactured plywood from compressed birch veneers and could consequently increase the bending and shear strength. Wang, et al. [33] were inspired by stiffness differences in the “sandwich structure” of earlywood and latewood. They demonstrated that a densification of the surface layers alone can enhance the bending properties and could increase the bending strength and modulus of elasticity by 54 and 104%, respectively. A comparison of the presented results with NFCs may be of additional help when evaluating the suitability of delignified densified plywood for high-performance applications. When analyzing a recently published Ashby-type material selection chart for a wide range of natural fiber reinforced polymer composites with different architecture [31], plywood produced in the present study performs similar to the top-range of multiaxial composites, whose modulus of elasticity ranges from 5 to 15 GPa, and strength from 80 to 150 MPa, and is even comparable to low-performance unidirectional composites.

**Figure 6:**
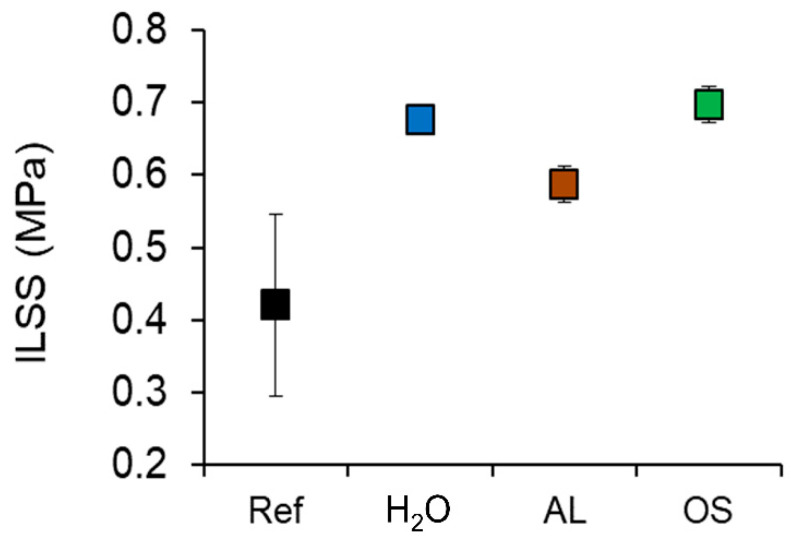
Mechanical characterization of plywood samples made of reference (Ref), densified water-soaked (H_2_O), densified alkali-treated (AL) and densified organosolv-treated (OS) spruce veneers conditioned at 20 °C and 65% relative humidity by interlaminar shear tests. (*n* = 6 for Ref; *n* = 6 for H_2_O; *n* = 6 for AL; *n* = 6 for OS).

Unfortunately, plywood produced from delignified and densified veneers also shows specific weaknesses. Bending strength is a combination of tensile strength, compression strength and shear strength, the latter being of particular relevance with short and thick specimens. Results of specimens with such geometry, yielding values of interlaminar shear strength, are shown in Figure 6. Overall, interlaminar shear strength does not follow the trend of increasing performance with increasing density to the same extent as is the case for the modulus of elasticity and bending strength. All plywood variants produced from densified wood show an improvement by a factor 1.4–1.6. For the variant H_2_O, this improvement factor is well in line with the improvement seen for modulus and bending strength, but for the delignified variants AL and OS, the improvement in shear strength is clearly below improvements in modulus and bending strength (roughly factor 3 and 2, respectively). Notably, the fracture pattern shows wood failure as opposed to adhesive failure for the variants AL and OS, suggesting a decrease in interfibrillar adhesion transverse to the fiber direction in delignified densified wood compared to untreated specimens.

**Figure 7:**
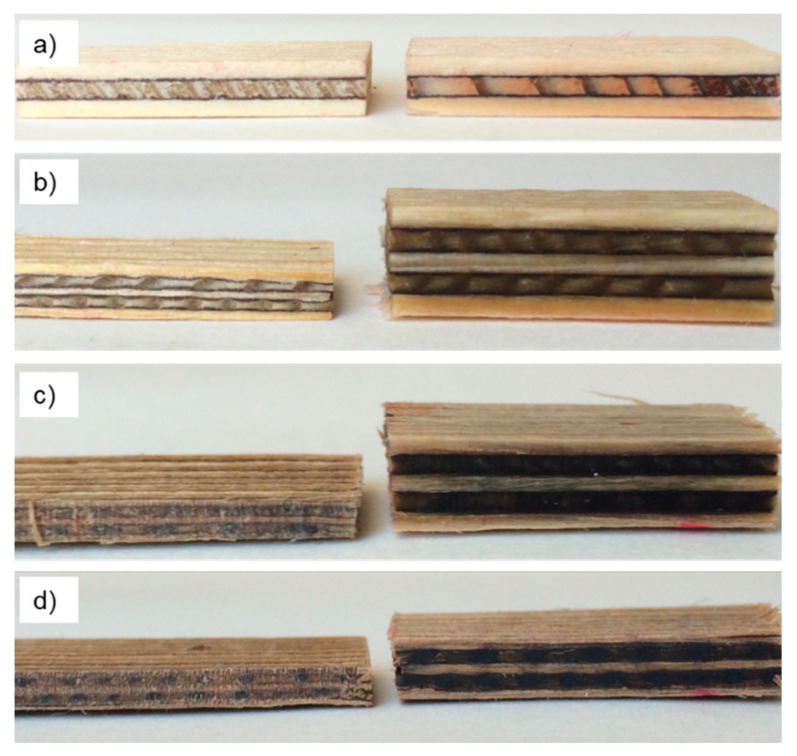
Representative photographs showing the swelling behavior of plywood samples: (**a**) made of reference veneers; (**b**) made of densified water-soaked veneers; (**c**) made of densified alkali-treated veneers; (**d**) made of densified organosolv-treated veneers. (Conditions: left = 20 °C, 65% relative humidity; right = after complete immersion in 20 °C deionized water for 24 h).

Table 4 and Figure 7 show the thickness swelling properties of the plywood samples after 24 h immersion in water. It is obvious that densified plywood tends to swell more than plywood made from untreated veneer. While plywood produced from untreated veneer exhibits only moderate swelling the variant H_2_O, which was densified after soaking in water only, almost doubles its thickness at full water saturation. Thus the densification is almost fully recovered in this variant, similarly to the variant AL, which exhibits even higher swelling. Surprisingly, the variant OS swells to a much smaller degree and thus experiences good stability even at high humidity. The reasons for the difference in swelling between densified AL- and OS-treated samples by a factor of 2.5, can only be speculated. It is well known, that the wood density influences its swelling behavior [34]. AL specimens show a higher density compared to OS specimens which may explain part of their increased swelling upon re-wetting. Furthermore, the calculated hemicelluloses content is higher for AL compared to OS (Table 2). Since hemicelluloses is the most hydrophilic constituent of the cell wall [35,36], this may also contribute to excessive swelling in AL specimens compared to OS.

## 4. Conclusions

Partial delignification and densification of spruce veneers results in a clear increase of mechanical performance. The improvement exceeds the pure scaling effect of increasing density and is also higher than the effect of densification without delignification. While stiffness improvement is also transferred to plywood produced from densified wood, strength improvement is less pronounced, possibly due to lack of interlaminar shear strength. Also, thickness swelling upon re-wetting was substantial. In conclusion, plywood from partially delignified and densified veneers is promising, but further work on adhesive bonding and prevention of thickness swelling is required. Nonetheless, a clear potential for competing with random-oriented or multiaxial woven fiber-reinforced polymers was identified.

## Figures and Tables

**Table 1:** Comparison of Klason lignin content and mass loss of native and chemically treated spruce veneers based on the assumption that no cellulose degradation occurs. (Klason lignin content: *n* = 2 for Ref; *n* = 2 for AL; *n* = 1 for OS. Mass loss: *n* = 4 for AL; *n* = 6 for OS).

Variant	Klason Lignin Content [%]	Mass Loss [%]	Klason Lignin Loss [%]	Hemicelluloses Loss [%]
Ref ^1^	28.6	-	-	-
AL ^2^	21.1	18.4 ± 0.03	11.4	7
OS ^3^	18.4	26.3 ± 0.04	15	11.3

^1^ Reference. ^2^ Alkali-treated. ^3^ Organosolv-treated.

**Table 2:** Comparison of chemical composition of native and chemically treated spruce veneers.

Variant	Cellulose [%]	Klason Lignin [%]	Hemicelluloses [%]
Ref ^1^	40.4 *	28.6	31 *
AL ^2^	49.5	21.1	29.4
OS ^3^	54.8	18.4	26.8

^1^ Reference. ^2^ Alkali-treated. ^3^ Organosolv-treated. * [26].

**Table 3:** Comparison of thickness and density of native, densified and densified chemically treated spruce veneers. (*n* = 5 for Ref; *n* = 5 for H_2_O; *n* = 5 for AL; *n* = 5 for OS).

Variant	Thickness [mm]	Density [g cm^−3^]
Ref ^1^	1.47 ± 0.02	0.39 ± 0.01
H_2_O ^2^	0.60 ± 0.04	0.78 ± 0.06
AL ^3^	0.51 ± 0.04	0.96 ± 0.07
OS ^4^	0.50 ± 0.05	0.83 ± 0.08

^1^ Reference. ^2^ Densified soaked with water. ^3^ Densified alkali-treated. ^4^ Densified organosolv-treated.

**Table 4:** Effect of densification and chemical treatments on the thickness swelling behavior of spruce plywood samples after complete immersion in 20 °C deionized water for 24 h. (*n* = 5 for Ref; *n* = 5 for H_2_O; *n* = 5 for AL; *n* = 5 for OS).

Variant	Number of Layers	Swelling [%]
Ref ^1^	3	8.3 ± 1
H_2_O ^2^	5	92.1 ± 7.5
AL ^3^	5	105.5 ± 5.1
OS ^4^	5	42 ± 2.1

^1^ Reference. ^2^ Densified water-soaked. ^3^ Densified alkali-treated. ^4^ Densified organosolv-treated.
